# Where Complexity Lives: Uncovering Clinical and Social Need Clusters in Acute Mental Health Services

**DOI:** 10.1192/bjo.2026.11824

**Published:** 2026-06-30

**Authors:** David Mirfin, Mark Pecover

**Affiliations:** Surrey and Borders Partnership NHS Foundation Trust, Guildford, United Kingdom

## Abstract

**Aims:**

Understanding how clinical and social needs cluster across acute mental-health services is essential for improving flow, reducing delays, and designing targeted pathways. We hypothesised that trauma, complex emotional needs (CEN), and mood/anxiety disorders would frequently co-occur across Surrey and Borders Partnership NHS Foundation Trust (SABP) acute pathways, and social-determinant burdens such as no fixed abode (NFA)/insecure housing and unmet social care needs would be concentrated in high-acuity services, including psychiatric intensive care units (PICU) and long length-of-stay (LLOS) populations.

**Methods:**

We conducted a cross-sectional audit of 99 service users across nine SABP populations including Safe Haven, Liaison Psychiatry, High-Intensity Users (HIU) in Home Treatment Team (HTT) and Liaison, HTT, inpatient wards, PICU, Health-Based Place of Safety (HBPoS), and LLOS. For each individual, binary coding captured clinical needs including frailty, psychosis/bipolar affective disorder, dementia, trauma, CEN, mood/anxiety disorders. Social determinants were also measured including substance use, offending/aggression, age 18–25, disordered eating, ethnicity non-white, NFA/insecure housing; social isolation; neurodevelopmental support; learning disability and unmet social care needs. We summarised prevalence and co-occurrence patterns across services.

**Results:**

Across the whole cohort, the most prevalent needs were mood/anxiety disorders 66.7%, CEN 60.6%, trauma 57.6%, substance use 55.6%, and neurodevelopmental support 50.5%. Co-occurrences were common including CEN + mood/anxiety disorders 50.5%, trauma + CEN 47.5%, and the trauma + CEN + mood/anxiety disorder triad in 42.4% of service users. Patterns varied across populations. HIU-HTT showed the highest combined clinical-need burden, with substance use 81.8%, mood/anxiety disorders 81.8%, CEN 72.7%, and trauma 72.7%. HIU-Liaison recorded 100% prevalence of trauma, CEN, and mood/anxiety with neurodivergence at 81.8%. As well as the highest percentage of psychosis/bipolar affective disorder 90.9%, PICU exhibited the greatest social-determinant burden, including NFA/insecure housing 27.3% and unmet social care needs 54.5%–the highest across all services. LLOS also showed elevated complexity, with neurodevelopmental support 81.8% and mood/anxiety disorders 72.7%. Safe Haven presentations were characterised by mood/anxiety disorders 90.9% and CEN 81.8%.

**Conclusion:**

Across nine acute-care populations, co-occurring trauma, CEN, and mood/anxiety disorders constitute the dominant clinical complexity patterns, while PICU and LLOS carry the heaviest burden of housing insecurity and unmet social-care need. These findings support targeted operational interventions including prioritising senior discharge coordination for PICU and LLOS and implementing regular multi-agency escalation huddles to address social-determinant drivers of delayed flow. This mapping demonstrates where complexity lives in the SABP system and provides actionable insights for service improvement.

